# Optical Biosensor Platforms Display Varying Sensitivity for the Direct Detection of Influenza RNA

**DOI:** 10.3390/bios11100367

**Published:** 2021-09-30

**Authors:** Samantha J. Courtney, Zachary R. Stromberg, Adán Myers y Gutiérrez, Daniel Jacobsen, Loreen R. Stromberg, Kiersten D. Lenz, James Theiler, Brian T. Foley, Jason Gans, Karina Yusim, Jessica Z. Kubicek-Sutherland

**Affiliations:** 1 Physical Chemistry and Applied Spectroscopy, Los Alamos National Laboratory, Los Alamos, NM 87545, USA; sjc@lanl.gov (S.J.C.); zrs@lanl.gov (Z.R.S.); djacobsen@lanl.gov (D.J.); loreen@lanl.gov (L.R.S.); kiersten@lanl.gov (K.D.L.); 2 Biosecurity and Public Health, Los Alamos National Laboratory, Los Alamos, NM 87545, USA; adanm@lanl.gov (A.M.y.G.); jgans@lanl.gov (J.G.); 3 Space Data Science and Systems, Los Alamos National Laboratory, Los Alamos, NM 87545, USA; jtheiler@lanl.gov; 4 Theoretical Biology and Biophysics, Los Alamos National Laboratory, Los Alamos, NM 87545, USA; btf@lanl.gov

**Keywords:** diagnostics, detection, biosensor, influenza, RNA, waveguide, flow cytometer

## Abstract

Detection methods that do not require nucleic acid amplification are advantageous for viral diagnostics due to their rapid results. These platforms could provide information for both accurate diagnoses and pandemic surveillance. Influenza virus is prone to pandemic-inducing genetic mutations, so there is a need to apply these detection platforms to influenza diagnostics. Here, we analyzed the Fast Evaluation of Viral Emerging Risks (FEVER) pipeline on ultrasensitive detection platforms, including a waveguide-based optical biosensor and a flow cytometry bead-based assay. The pipeline was also evaluated in silico for sequence coverage in comparison to the U.S. Centers for Disease Control and Prevention’s (CDC) influenza A and B diagnostic assays. The influenza FEVER probe design had a higher tolerance for mismatched bases than the CDC’s probes, and the FEVER probes altogether had a higher detection rate for influenza isolate sequences from GenBank. When formatted for use as molecular beacons, the FEVER probes detected influenza RNA as low as 50 nM on the waveguide-based optical biosensor and 1 nM on the flow cytometer. In addition to molecular beacons, which have an inherently high background signal we also developed an exonuclease selection method that could detect 500 pM of RNA. The combination of high-coverage probes developed using the FEVER pipeline coupled with ultrasensitive optical biosensors is a promising approach for future influenza diagnostic and biosurveillance applications.

## 1. Introduction

Influenza is a rapidly evolving viral pathogen that infects up to 5 million people annually [1]. There are four viral influenza genera: A, B, C, and D [2]. Human infections are most commonly caused by influenza A (IAV) and B (IBV) viruses [3]. IAV has caused four major pandemics (in 1918, 1958, 1968, and 2009) [4]. Pandemics occur when a new viral strain evolves through genome reassortment producing influenza variants for which there is no pre-existing human immunity [4,5,6]. These novel variants often emerge from bird or pig reservoirs [7]. For timely and effective treatment and to halt the spread of new pandemic variants, a rapid method for identifying novel viruses and diagnosing infected patients is needed [8]. A method for rapid and accurate point-of-care viral diagnostics, both for influenza and other pathogens, would provide time to implement prevention measures and to rule out pandemic infections so that patients receive proper medical treatment [9].

The traditional (“gold standard”) method for influenza diagnostics is viral culture, in which permissive cell lines are inoculated with a patient sample to see if influenza virus propagation occurs within 10 days [10]. This method is extremely reliable but does not provide a timely result to guide antiviral administration or quarantine procedures. As a consequence, other diagnostic methods are more widely used to inform patient treatment, including rapid influenza diagnostics tests (RIDTs) and real-time polymerase chain reaction (RT-PCR). RIDTs detect specific influenza surface antigens within 15 min with sensitivities ranging from 10–70% [10]. This low sensitivity results in an unacceptably high rate of undiagnosed infections [10]. Nucleic acid detection assays for influenza provide a promising enhancement in the sensitivity of influenza diagnostics [11]. There are a variety of nucleic acid-based techniques approved by the FDA for the detection of influenza, most of which are based on PCR technology [11]. PCR relies on exponential enzymatic amplification of a specific target nucleic acid sequence [12]. The most rapid of these technologies include Abbott’s ID NOW (15 min), Cepheid’s GeneXpert (30 min), and BioFire Diagnostics FilmArray (2 h). However, these tests have shown an inability to reliably detect circulating IAVs, let alone other influenza genera, due to virus variability in the target region [11,13]. To reduce the false-negative rate, mismatch-tolerant single-stranded DNA molecular beacons (MBs) have been used to detect influenza viruses of both animal and human origin [13]. These short oligonucleotide probes are comprised of hairpin structures labeled with a quencher and fluorophore. However, this strategy still requires PCR amplification of a target sequence. Thus, there is an urgent need for diagnostic tests that are highly sensitive and display broad coverage for point-of-care and surveillance efforts [10].

Nucleic acid-based sensors have been reported to detect 10^−18^ M nucleic acid target concentrations using optical or electrochemical techniques without PCR amplification and have the ability to provide high-throughput data for surveillance [8]. However, sensitivity and specificity can be significantly altered in complex biological samples. There are several versions of ultrasensitive biosensor immobilization techniques and signal detection, including optical and piezoelectric surfaces for nucleic acid detection [14,15,16,17,18,19]. Biosensor surface chemistry allows nucleic acid probes to anchor to its surface; when viral RNA is introduced to the biosensor, the complimentary probes bind to RNA with varying sensitivity and specificity [20]. These platforms can have a low limit of detection and rapid sensing capabilities [21]. Previously, we described the development of a waveguide-based optical biosensor that can detect lipid and amphiphilic pathogen biomarkers [22,23]. Here, this waveguide-based optical biosensor was used to detect influenza RNA. This platform was compared to flow cytometry detection for the direct detection of influenza nucleic acids, which has been reported to detect viral DNA or RNA down to the femtomolar level [24].

Our nucleic acid detection probes for these biosensor platforms utilized MBs, as assay format that has been explored previously in other biosensor platforms for the amplification-free detection of influenza RNA [15,25]. MBs are sensitive and can be both highly specific to their target, as well as mismatch tolerant to detect multiple influenza strains with diverse sequences [26,27]. This work presents the development of novel, algorithmically designed, high-coverage probe sequences and explores their feasibility for amplification-free influenza detection in a thermal cycler, waveguide-based optical biosensor and flow cytometer instrument.

## 2. Materials and Methods

### 2.1. Design and Synthesis of FEVER MB Probes and Respective Synthetic Targets

MB probes were designed using the Fast Evaluation of Viral Emerging Risks (FEVER) computational pipeline developed at Los Alamos National Laboratory (LANL) [28]. Our probes target the highly conserved genome segment 3 encoding the polymerase (PA) gene. We designed probes based on a multiple sequence alignment set derived from the GISAID database (with sequences of samples from 1918 to August 2019). Redundancy in the alignment was reduced by removing identical sequences, which left 58,706 influenza A virus sequences and 9202 influenza B virus sequences that were used to design the IAV and IBV probes. The FEVER algorithm designed probes by identifying the most highly conserved regions in IAV and IBV sequences. One IAV and one IBV probe sequence was selected for testing. In addition to a high degree of sequence conservation, we also screened for low hairpin propensity, length of 30–35 bp, and GC content of at least 50%. Once the probe sequence was designed, a complementary 6 base pair stem sequence was added (CGCGAT) to both the 5′ (5′-CGCGAT-3′) and 3′ (5′-ATCGCG-3′) ends, along with a 5′ fluorophore Alexa Fluor 532 (AF532) and 3′ quencher BHQ-1. The 3′ stem region also incorporates a biotin-modified thymidine residue. DNA probes were synthesized by Integrated DNA Technologies Inc. (IDT, Coralville, IA, USA). Additionally, two synthetic RNA targets were synthesized by IDT that either perfectly matched the probe sequence or contained one to two mismatched bases from the probe sequence (Table 1). The single mismatched bases were chosen as the single most prevalent in the IAV and IBV GISAID sequences.

### 2.2. In Silico Inclusivity Test

Assay designs were computationally characterized using an in silico assay validation tool [28] to assess the inclusivity of the FEVER and U.S. CDC assay oligonucleotides. Each assay oligonucleotide was searched against influenza sequences obtained from GenBank (29–30 March 2021). GenBank sequences were filtered by length to remove any incomplete sequence fragments with the following, segment-specific length thresholds. For segment 3 (targeted by FEVER_IAV and FEVER_IBV), GenBank sequences with less than 2000 bases were excluded. For segment 7 (targeted by U.S. CDC Flu A), GenBank sequences with less than 900 bases were excluded. For segment 8 (targeted by U.S. CDC Flu B), GenBank sequences with less than 800 bases were excluded. In silico search results were quantified using the recall (=true-positive rate = (true positives)/(true positives + false negatives)). False-negatives were defined as either (a) 3 or more mismatches or a predicted melting temperature less than 40 °C for the oligonucleotide and target genome sequence, or (b) when mismatches occurred in the last two 3′ bases of a primer that resulted in a threshold cycle (C_T_) increase of 2 more as defined previously [29]. True positives were defined as any pairing that did not result in one of these conditions being met. Results were also reported as the number of genome sequences that, when paired with an assay oligonucleotide, resulted in either a mismatch, two mismatches, a perfect match, or a failure (false negative).

### 2.3. FEVER MB Probe-RNA Hybridization Thermodynamics

The hybridization thermodynamics of FEVER MB probes A and B to their respective synthetic RNA targets were experimentally characterized on the StepOne Plus Real-Time PCR System (Applied Biosystems, Waltham, MA, USA) using the following melt curve conditions: holding stage at room temperature (25 °C) for 5 min, melt curve stage step and hold (26 °C to 95 °C) with 5 °C increments/min. Fluorescence intensity was measured for probes alone (200 nM) and probes mixed at a 1:4 probe to synthetic target ratio (200 nM probe: 50 nM synthetic target, unless otherwise stated).

### 2.4. Waveguide-Based Optical Biosensor Detection

Biosensor experiments were carried out using a waveguide-based optical biosensor developed at LANL as previously described [23,30,31]. Lipid bilayers were prepared [32] and deposited on planar silicon oxynitride (SiONx) optical waveguides (nGimat Ltd., Atlanta, GA, USA) coated with a 10 nm surface of SiO_2_ (Spectrum Thin Films Inc., Hauppangeg, NY, USA). Waveguides were cleaned and lipid bilayers were prepared as described previously [33]. Lipids containing 1% biotin were made from 60 µL of 5 mM 1,2-Dioleoylsn-glycero-3-phosphocholine (DOPC) and 0.6 µL of 5 mM 1,2-dioleoyl-sn-glycero-3-phosphoethanolamine-N-(cap biotinyl) (Avanti Polar Lipids, Alabaster, AL, USA), rehydrated in 600 µL Dulbecco’s phosphate-buffered saline (PBS, D8662, Millipore Sigma, St. Louis, MO, USA), subject to 10 freeze/thaw cycles in liquid nitrogen, and then probe sonicated for 6 min (1 s pulse on, 1 s pulse off). The assay flow cell was assembled by securing a waveguide to a glass coverslip with two holes drilled for inflow and outflow (Thermo Fisher Scientific, Waltham, MA, USA) with a silicone gasket containing a laser cut channel to create a flow cell (Grace Bio-Labs, Bend, OR, USA). In total, 60 µL of lipids were added to the flow cell and incubated at 4 °C overnight to allow bilayer formation.

All washes and incubations occurred at room temperature. The lipid bilayer was blocked with 2% bovine serum albumin (BSA, A7906, Millipore Sigma, St. Louis, MO, USA in PBS for 1 h, and all washes were performed with 2 mL of 0.5% BSA in PBS. All other injections were 100 µL volumes, and all dilutions were made in PBS unless otherwise specified. The lipid bilayer integrity was determined by injecting 1 nM streptavidin conjugated to AF532 and incubating for 5 min. This signal was photobleached, and then 1 µM unlabeled streptavidin was injected and incubated for 10 min. Two concentrations of exact match RNA (50 nM or 100 nM, and 1 µM) were tested by pre-incubating with 100 nM of biotinylated IAV MB for 10 min to allow hybridization and then injected and incubated for an additional 10 min to allow binding of the biotinylated probe + RNA complex to the streptavidin surface. For experiments with saliva, capture probe and viral RNA (100 nM and 1 µM) were diluted and pre-incubated in normal human saliva pooled from at least 3 donors (Lee Biosolutions, cat. no. 991-05-P) instead of PBS. The surface was washed and the specific signal was measured. Spectra were obtained using a neutral density 1.0 filter to minimize the signal observed by the probe alone. Inherent waveguide differences were accounted for by normalizing raw fluorescent intensity data by dividing the probe alone or probe + RNA spectra by the streptavidin-AF532 spectra.

### 2.5. Flow Cytometry Bead-Based Detection of Synthetic Influenza A Targets

Streptavidin-coated polystyrene particles (6–8 µm diameter, Spherotech, Inc., Lake Forest, IL, USA) were washed twice in PBS (Millipore Sigma, St. Louis, MO, USA) and resuspended at 10^6^ particles/mL in PBS. Then, 20 µL of streptavidin-coated polystyrene particles were incubated with 10 µL biotinylated IAV FEVER MB probe (200 nM) for 5 min in the dark with gentle agitation. Streptavidin-coated polystyrene particles and biotinylated probe were then washed twice to remove any unbound probe and resuspended in 100 µL PBS. Serial dilutions of the synthetic IAV RNA match target (10 nM to 100 fM RNA) were separately incubated with the streptavidin-coated polystyrene particles and probe for 10 min in the dark with gentle agitation. The streptavidin-coated polystyrene particles, probe, and target were washed twice and resuspended in 200 µL PBS, where the final bead concentration was 10^4^ streptavidin-coated polystyrene particles/test. This protocol was modified from methods previously described by Horejsh et al. [24]. Samples were analyzed using a CytoFlex S flow cytometer (Beckman Coulter Life Sciences, Indianapolis, IN, USA) equipped with 405 nm, 488 nm, 561 nm, and 638 nm lasers and appropriate filters. The cytometer was calibrated prior to the experiment by running Daily QC Fluorospheres per manufacturer recommendations. Gains on the FITC channel were adjusted experimentally to maximize signal to noise ratios. Data sets were evaluated using FlowJo 10.7.1. by importing FSC files and running the sample quality check feature to ensure sample collection uniformity. Gating strategies were developed first by visualizing forward and side-scatter area (FSC-A vs. SSC-A) plots and then gating single particles on an SSC-H vs. SSC-A plot of the streptavidin bead control. To visualize the fluorescence shift between the negative (no RNA) and positive control samples, the singles gate was applied to all samples, and the 532 nm probe fluorescence was collected and displayed in the FITC channel as FITC-A versus SSC-A pseudocolor dot plots for 20,000 events. To confirm fluorescence peaks, plots were transformed to histograms, and FITC (+), FITC (−), and FITC (q) gates were identified using FlowJo (Figure S5).

### 2.6. Exonuclease Selection

The same IAV and IBV FEVER probe target sequences used for MBs (Table 1) were used to develop exonuclease selection assays excluding stem sequences, fluorophore and quencher. Probes were synthesized by IDT (Table 2). CP_IAV_Exo is the capture probe, CP_fwd and CP_rev were forward and reverse primers, respectively for PCR, and Rep_F3_IAV is the reporter probe used in flow cytometry experiments. For experiments, 10 nM probes and synthetic IAV match RNA were mixed together in 10 µL 1X NEB 3.1 buffer and incubated at room temperature for 15 min. For experiments with saliva, 10-fold dilutions of capture probe and viral RNA were performed in normal human saliva pooled from at least 3 donors (Lee Biosolutions, cat. no. 991-05-P). Then, 20 U Thermolabile Exonuclease I (NEB M0568L) was added and volume brought to 20 µL with 1X NEB 3.1 buffer and incubated at 37 °C for 30 min. After single-stranded DNA probe is digested, probes hybridized to RNA target were incubated with 200 nM reporter probe and at the same time bound to streptavidin-coated polystyrene particles as described in Section 2.5. Negative control samples were treated identical to test samples, but did not contain any synthetic IAV match RNA. Probe remaining after exonuclease digestion was measured via PCR using the primers in Table 2.

### 2.7. Statistical Analysis

Data were analyzed and visualized using GraphPad Prism version 9. *p* values of <0.05 were considered significant. Flow cytometry statistical data on the FITC gates was analyzed on FlowJo 10.7.1.

## 3. Results

### 3.1. In Silico Inclusivity Test against Influenza Sequences

The recall, hybridization efficiency, and mismatch tolerance of the IAV and IBV FEVER probe sequences were compared to the CDC’s probes (Table 3). The associated CDC primer sequences were not included in this comparison. For both IAV and IBV, the FEVER probe sequences (IAV, 96.2% recall; IBV, 99.6% recall) performed better in recall than the CDC probe sequences (IAV, 91.3% recall; IBV, 97.9% recall). Although the IAV and IBV FEVER probes detected a relatively fewer percentage of perfect match sequences in silico as the CDC IAV and IBV probes, they detected a relatively greater percentage of mismatch sequences with one or two mismatched bases (IAV: 18,615 one mismatch sequences and 2804 two mismatch sequences; IBV: 1618 one mismatch sequences and 339 two mismatch sequences). In addition, the IAV and IBV FEVER probes had a predicted lower percentage of false negatives (sequences that failed) compared to the CDC probes (IAV 3.8% vs. 8.7%, respectively; IAB, 0.4% vs. 2.1%, respectively).

### 3.2. RNA Detection Using Molecular Beacon Probes

#### 3.2.1. Thermal Cycler

**Figure 1:**
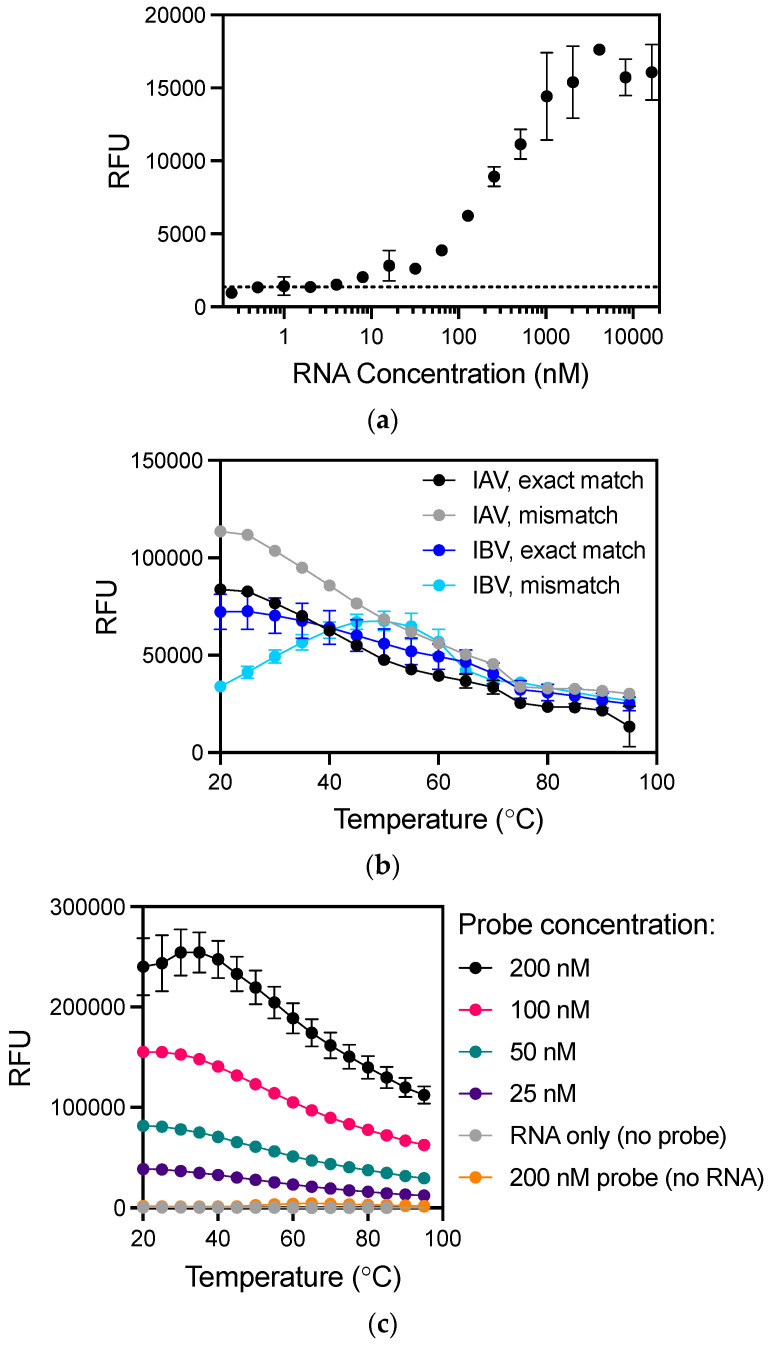
Direct detection of influenza RNA without amplification using a thermal cycler. (**a**) The lowest concentration detected by 100 nM of the IAV probe with exact match RNA at room temperature was 8 nM (*p*  <  0.05). Dotted line indicates background signal. (**b**) Mismatch tolerance of MB probes was determined using synthetic RNA with exact match sequence and a single mismatch (Table 1). All targets were detected above probe background noise (*p* < 0.0001 for match A, mismatch A, match B, mismatch B). (**c**) Hybridization kinetics at varying IAV MB probe concentrations (0–200 nM) was determined using 50 nM synthetic RNA exact match target with even the lowest 25 nM probe concentration sufficient to detect 50 nM RNA over background noise (*p* < 0.0001). Values plotted are mean ± standard deviation. Statistical significance was determined by Student’s *t*-test for (**a**–**b**) and by two-way ANOVA with Dunnett’s multiple comparisons test to determine individual variances for (**c**). RFU, relative fluorescence units.

To establish a baseline and optimize hybridization kinetics, the sensitivity of a thermal cycler to directly detect influenza RNA without PCR was analyzed by varying either the probe or target RNA concentration (Figure 1). To enable direct comparison of results between assay platforms, a single temperature (25 °C) similar to all platforms tested was investigated. The FEVER IAV MB probe was incubated with a range of RNA concentrations at room temperature (25 °C). The lowest concentration detected by 100 nM of the FEVER IAV MB probe was 8 nM of exact match RNA (Figure 1a). To evaluate the prediction that FEVER influenza probes would generate fewer false negative results by allowing mismatches, the FEVER IAV and IBV probes were experimentally evaluated for their mismatch tolerance at room temperature. Both detected the RNA targets with a significant signal to background noise ratio (*p* < 0.0001 by *t*-test), where fluorescent values were normalized to the probe background noise (Figure 1b). Mismatch tolerance did not alter the specificity of the IAV probe to detect up to 1 µM of IBV RNA at any temperature between 25 and 95 °C, and the same was true for the IBV probe with IAV RNA (Figure S2). PBS was selected as the optimal buffer condition for probe and target hybridization since fluorescence intensity was highest when compared to a variety of other solutions (Figure S1). Next, the fluorescent signal as a function of probe concentration was analyzed by decreasing IAV FEVER MB probe concentrations in the presence of 50 nM synthetic influenza RNA (Figure 1c). Together these results show that although RNA can be detected at room temperature using MB probes and a thermal cycler for imaging, the detection limit is poor and requires optimization beyond hybridization conditions and probe or target concentrations.

#### 3.2.2. Waveguide-Based Optical Biosensor

**Figure 2:**
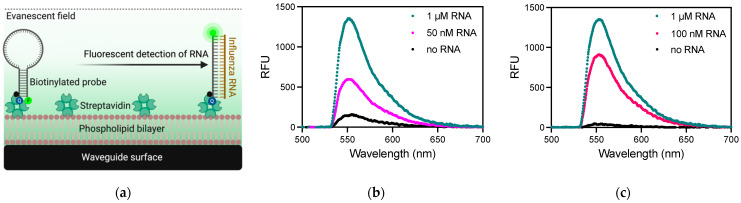
Direct detection of RNA using a waveguide-based optical biosensor. (**a**) Schematic of functionalized waveguide surface using a phospholipid bilayer and streptavidin to capture a biotinylated molecular beacon probe where fluorescence is quenched until hybridization with influenza RNA occurs (not drawn to scale). The fluorophore is excited by the evanescent field emitted from total internal reflection of light coupled in the waveguide limiting detection in this system to surface-bound molecules. Q, quencher; F, fluorophore. Figure created with Biorender.com. (**b**) Measurement of 50 nM (light pink line) and 1 µM (teal line) IAV RNA from the detection of AF532-labeled FEVER MB probe as compared to the quenched probe alone in the absence of RNA target (black line) in 1X PBS. (**c**) 100 nM (dark pink line) and 1 µM (teal line) RNA was also detected directly in human saliva. RFU, relative fluorescent units.

The LANL waveguide-based optical biosensor has been used previously to detect bacterial [34,35,36] and viral [37] pathogens, toxins [31], and tumor markers [23,38]. This is the first report of RNA detection using this optical biosensor platform. All incubations were performed at room temperature (between 20 and 25 °C). The waveguide surface was functionalized using a phospholipid bilayer intercalated with biotin, which allows for the use of biotin-streptavidin chemistry to capture the biotinylated MB probes (Figure 2a). The non-specific background signal is measured with the probe only, in the absence of target RNA, which quantifies the incomplete quenching of the fluorophore. For 100 nM probe only, this non-specific signal saturated in the biosensor, so all subsequent measurements were taken using an in-line 1.0 ND filter to reduce the signal to ~150 relative fluorescent units (RFU). The specific signal observed in PBS following the incubation of 100 nM MB probe with 50 nM or 1 µM synthetic IAV RNA was recorded in RFU, with maximum signals at 556 nm of 575 RFU and 1304 RFU, respectively. The waveguide-based optical biosensor was able to detect 50 nM RNA with a signal/noise of 3.66 ± 0.04. The sensitivity of this assay was also tested in human saliva to assess feasibility in a complex biological sample. The specific signal observed in saliva with 1 µM synthetic IAV RNA was 1331 RFU (Figure 2c), which was nearly identical to the signal observed in PBS showing that saliva did not significantly affect the direct detection of RNA in the waveguide-based optical biosensor platform. The sensitivity of this platform to detect RNA using MB probes is limited by the high fluorescence observed from the incomplete quenching of the fluorophore in the probe-closed conformation (no template RNA). Different incubation temperatures and alternative probe formats should be explored to minimize non-specific background fluorescence observed in this surface-based optical measurement format.

#### 3.2.3. Flow Cytometer

**Figure 3:**
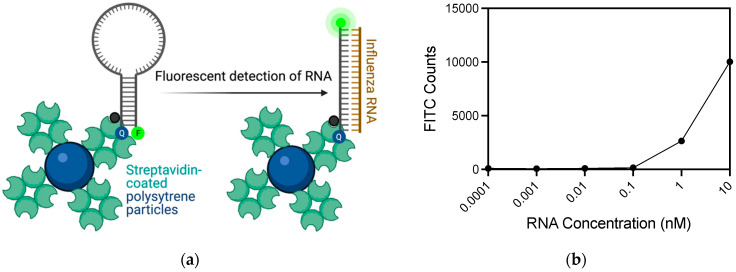
Flow cytometry bead-based detection of influenza RNA. (**a**) Schematic of flow cytometry bead-based assay using streptavidin-coated polystyrene particles coated with biotinylated MB probes. In the absence of target RNA fluorescence is quenched, but when target is present the fluorophore is separated from the quencher and fluorescence resulting from excitation is measured in the FITC channel of the flow cytometer. Figure created with Biorender.com. (**b**) The flow cytometer was able to detect 1 nM IAV RNA with 200 nM MB. The fluorescent signal from MB-RNA hybridization was quantified by FITC counts derived from the mean of 20,000 events.

A flow cytometry assay was developed as another platform for IAV RNA detection with the IAV FEVER MB probe (Figure 3a). Biotinylated probes were incubated with streptavidin-coated polystyrene particles. When complementary, synthetic IAV RNA was introduced to the aqueous solution of streptavidin-coated particles with attached MB probes, probe-target hybridization occurred separating the probe’s BHQ-1 quencher from the AF532 fluorophore, causing fluorescence to emit when excited. Fluorescent signal was detected in the FITC channel of the flow cytometer, which was optimized using both RNA and DNA targets with exact match and single mismatch target sequences (Figure S3, Table S1). Data processing and gating strategies were optimized as described (Figure S4). At 10 nM RNA, 50% of the events fell within the probe + RNA gate, indicating distinct fluorescence from MB-RNA hybridization. However, many (38.8%) of the counts fell within the quenched (probe-only) gate with a weaker fluorescence signal from the MB-RNA hybridization (Figure S4). The assay was tested with serial dilutions of synthetic IAV match RNA from 10 nM RNA down to 100 fM (Figure 3b). The probe + RNA gate shows an apparent decrease in signal counts after 1 nM RNA. The probe-only FITC counts increase as the RNA concentration decreases (Figure S5), indicating that the fluorescent signals from MB-RNA hybridization under 1 nM RNA is shifted left into the probe-only gate. This indicated an attenuated signal due to lower concentrations of RNA. However, the probe-only gate is only indicative of diminished fluorescence, which would be expected as fewer MB hairpin probes are forced open when lower concentrations of RNA are present. Lower fluorescence counts in the probe + RNA gate and higher counts in the probe-only gate indicate the assay is still positive even at lower concentrations of RNA. Therefore, the lowest concentration detected by MB-RNA hybridization on the flow cytometer was 1 nM RNA.

### 3.3. RNA Detection Using Exonuclease Selection

**Figure 4:**
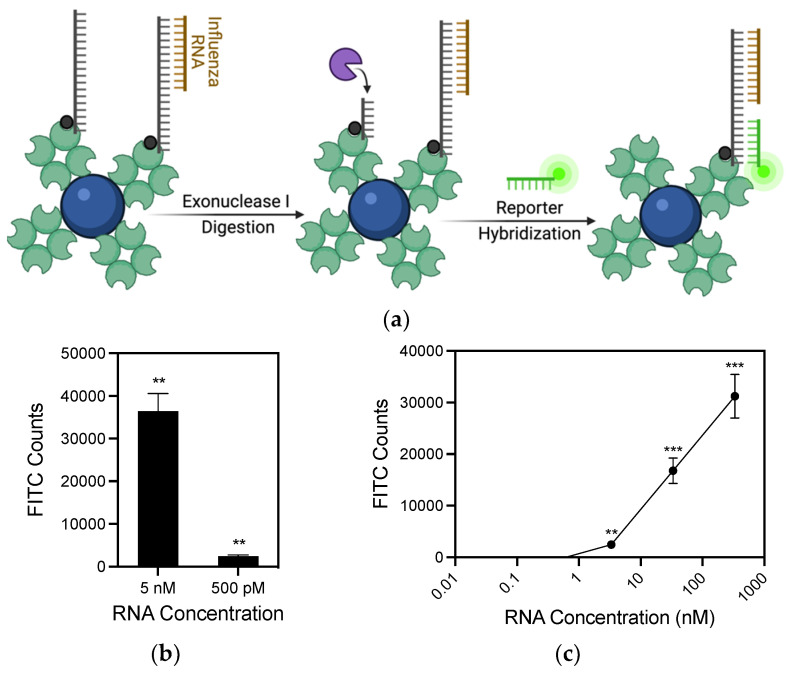
Viral RNA detection using exonuclease selection. (**a**) Schematic of the exonuclease RNA detection strategy. Probe bound with viral RNA is not digested by ExoI. After digestion, remaining probes are hybridized with complementary reporter probes containing a single fluorophore. Figure created with Biorender.com. (**b**) 500 pM RNA was detected using 10 nM probes. (**c**) 3 nM RNA was detected directly in human saliva. Values plotted are the mean ± standard deviation. Statistical significance of FITC counts with RNA present compared to no RNA control was determined by Student’s *t*-test (** *p* < 0.01, *** *p* < 0.001).

One limitation of MBs is their high background fluorescent signal. MBs rely on efficient quenching from closed hairpins, which is often incomplete and detected using ultrasensitive optical sensors. To reduce background signal and enhance the sensitivity of RNA detection, an alternative method using exonucleases was developed (Figure 4a). Novel probes were created containing the same FEVER IAV sequence at the 3′ end, with a 5′ biotin and a middle linker sequence (Table 2). When IAV RNA binds to the 3′ region of the probe, Exonuclease I (ExoI) is added to selectively digest only remaining unbound single-stranded DNA probes leaving only the bound probes. After digestion and washing away of the exonuclease, a fluorescent probe is added that binds the middle linker sequence of the probe. A flow cytometry experiment was used to compare the exonuclease selection to MBs. The results show a significant signal over background at 500 pM (Figure 4b). Lower amounts may still be detected, as measured by the amount of probe remaining after exonuclease digestion when comparing samples against probe digested with no synthetic IAV (Figure S6). In order to test the efficacy of this assay in a complex biological sample, 10-fold serial dilutions were made in human saliva (Figure 4c). In saliva, as low as 3 nM RNA was detected showing a reduction in sensitivity by less than an order of magnitude in the absence of any sample processing, which could be optimized to further enhance assay sensitivity.

## 4. Discussion

Early detection of influenza is critical to prevent viral spread between individuals before or during an outbreak, and to ensure that influenza infections can be differentiated from other infectious illnesses. Methods to detect and differentiate between viral strains must be rapid, accurate, sensitive and deployable at the point-of-need. Turn-around time for commercial RT-PCR assays for IAV detection can range from 20 min to over 2 h with the amplification step taking up the majority of the time [39]. Alternative methods are being developed to overcome these hurdles at the point-of-need with low cost, portability, ease of use and rapid results [40,41]. Here, we demonstrated the feasibility of two optical biosensors for influenza detection using molecular beacon probes designed with our FEVER pipeline. Typically, optical biosensors detect nucleic acids either by changes in visual characteristics when a target binds with a probe, or by labelling the probe with a fluorophore that gives a spectrally detectable signal as demonstrated in the current study [42]. Overall, coupling a method for improved molecular probe design with an optical biosensor is a promising initial step toward direct detection of influenza RNA.

The success of optical biosensors to detect nucleic acids can be enhanced by highly conserved, mismatch tolerant probes. We used our FEVER approach that has previously been applied to COVID-19 diagnostics, to design high-coverage MB probes [43]. Before testing experimentally, the MB probes were computationally evaluated compared to the benchmark U.S. CDC influenza probes as a benchmark. The inclusivity test determined that the IAV and IBV FEVER MB probes had a higher overall predicted recall and mismatch tolerance than the CDC’s IAV and IBV PCR probes. Ultimately, the FEVER MB probes could be more competitive in the diagnostic field in terms of sequence coverage and pandemic surveillance.

IAV and IBV have an estimated evolutionary rate of 2.6 × 10^−3^ and 0.5 × 10^−3^ mutations per site per year, respectively [44]. Molecular probes must tolerate mutations to detect genetically diverse influenza viruses [13]. The thermodynamic studies of MB-RNA hybridization elucidated that the IAV and IBV FEVER MB probes tolerated common mismatch sequences, confirming the results from the in silico analysis. There was no decrease in relative fluorescence between the exact and mismatch target for IAV and a slight decrease in relative fluorescence between the match and mismatch target for IBV. This experimental data supports the in silico observation of high coverage for the IAV and IBV FEVER MB probes. In total, 8 nM of target RNA was detected without amplification on a thermal cycler. To improve the sensitivity of detection, we evaluated alternative biosensing strategies and probe formats.

Previously, intact influenza virus was detected on the LANL waveguide-based optical biosensor [37]. However, intact influenza virus may not be accessible or stable, depending on the stage of infection, while extracted RNA is commonly used for screening respiratory viruses [45]. Here, we showed that the waveguide-based optical biosensor was able to detect 50 nM RNA, which was not an improvement in sensitivity over using a thermal cycler. However, the low cost, portability and ease of use of the waveguide-based sensor make this system a promising method for directly detecting viral RNA at the point of need with further optimization. The limiting factors in these waveguide-based assays are (1) the high background fluorescence observed from the closed probe conformation in the absence of RNA, and (2) diffusion of the biotinylated probes to the immobilized streptavidin coated surface [46].

We transitioned our MB probes from the surface-based waveguide assay to a solution-based flow cytometry assay. The flow cytometer assay displayed enhanced sensitivity over both the waveguide sensor and thermal cycler by detecting 1 nM IAV RNA using MB probes. In these assays we were able to account for the high background of probe-only (no RNA target) through data analysis and gating strategies, and unlike the waveguide sensor which takes single measurements, the flow cytometer samples thousands of events for much higher throughput. We showed that FEVER probes are compatible with a flow cytometer sensing strategy and could be applied with minimal difficulty to the diagnostic field [47]. In addition, flow cytometry is widely available in clinical settings [48]. However, the overlap in signal between the probe + RNA gate and the probe-only gate reduces the assay sensitivity.

In order to reduce the background noise associated with incomplete quenching of the MB fluorophore, we explored an alternative probe design strategy using exonuclease selection. Exonuclease selection has been recently used in biosensors as a means of reducing background noise, as well as for target amplification [49,50,51]. A wide range of exonucleases allows for selecting desired properties, such as optimal reaction temperature and whether bound or unbound molecules are removed. Exonuclease selection is frequently coupled with aptamers, nanoparticles, and with binding of molecules such as transcription factors to DNA [52]. This work uses a system with only nucleic acids and exonucleases, which removes the need for complex design procedures such as selecting aptamers [50,51,53,54] or DNAzymes [55,56], designed nanoparticles [49], or fabricated electrodes [57]. This simple system is compatible with a wide range of optical sensors, including qPCR thermocyclers, flow cytometry, and the optical biosensors mentioned in this work. On the flow cytometer, the exonuclease selection detected RNA at 500 pM, well below the lowest amount detected for molecular beacons in this study.

Overall, the flow cytometer detected influenza RNA at lower levels than the thermal cycler and waveguide-based optical biosensor. Future studies are needed to optimize sensitivities to achieve lower levels of detection without amplification as well as improve probe to target hybridization to reduce time-to-result. In addition, the approach of coupling the mismatch tolerant FEVER probe design with an optical biosensor could be used for rapid screening of other RNA viruses such as HIV, Ebola, Zika, and SARS-CoV-2.

## 5. Conclusions

The IAV and IBV FEVER probes are predicted to detect influenza RNA with a high mismatch tolerance needed for future biosurveillance and diagnostic applications. The FEVER MB probes detected influenza RNA directly without nucleic acid amplification on both a waveguide-based optical biosensor (≥50 nM) and a flow cytometer (≥500 pM). The high-coverage probes developed using the FEVER pipeline are compatible with alternative probe formats including exonuclease selection as well as other aqueous- and surface-based detection platforms. This initial proof-of-concept study demonstrates the potential for improved sensitivity for the direct detection of viral RNA using ultrasensitive biosensors, which could facilitate rapid point-of-care diagnostic technologies. Future studies will aim to utilize these methods for direct detection or viral RNA in patient samples including saliva and nasal swabs. In summary, optical biosensing platforms, combined with high-coverage FEVER probe design, provide a promising avenue for rapid viral diagnostics and biosurveillance applications that can be optimized for use at the point of need.

## Figures and Tables

**Table 1:** Sequences of FEVER influenza MB probes and the synthetic RNA match and single base pair mismatch target sequences.

FEVER Probe	FEVER Probe Sequence	RNA Target Sequences
IAV	5′-/5Alexa532N/CGCGATGAGGAGTGCCTGATTAATGATCCCTGGGTTTA/BiodT/CGCG/3BHQ-1/-3′	Match: UAAGCAAAACCCAGGGAUCAUUAAUCAGGCACUCCUCAAUUGC (13,710.3 g/mol)
		Mismatch: UAAGCAAAACCCAGGGAUC**G**UUAAUCAGGCACUCCUCAAUUGC (13,726.3 g/mol)
IBV	5′-/5Alexa532N/CGCGATGAGGGAATGCCAAGAACCATAGCATGGATGGA/BiodT/CGCG/3BHQ-1/-3′	Match: UUUGGACCAUCCAUGCUAUGGUUCUUGGCAUUCCCUCAAUUAC (13,549 g/mol)
		Mismatch: UUUGGACCAUCCAUGCUAUG**U**UUCUUGGCAUUCCCUCAAUUAC (13,510 g/mol)

Stem region of probes is underlined. The single mismatch base of the mismatch RNA target sequences is bold.

**Table 2:** Sequences of FEVER influenza exonuclease selection probes.

Probe Name	Probe Sequence
CP_IAV_Exo	/5Biosg/GGCTTCAAGGAACGAG TCATTGGTGTTCGCGAACTGGGTAGTATCGAGCGCTGTGAACATCGGAGGAGTGCCTGATTAATGATCCCTGGGTTT
CP_fwd	GAGTCATTCCCGACCGTACTATGATAC
CP_rev	CGTTGTTGCACGAGGGTACTAC
Rep_F3_IAV	/5Alex532N/CGCTCGATACTACCCAGTT*C*G

An asterisk (*) indicates a phosphothiorate bond.

**Table 3:** Inclusivity test comparing FEVER and CDC IAV and IBV probes.

Assay	Recall ^a^	Total No. Sequences Analyzed	No. Sequences with Perfect Match	No. Sequences with 1 Mismatch	No. Sequences with 2 Mismatches	No. Sequences that Failed ^b^
FEVER_IAV	96.21%	73,854	49,634	18,615	2,804	2,801
U.S. CDC IAV	91.30%	85,087	66,614	10,544	524	7405
FEVER_IBV	99.58%	11,507	9502	1618	339	48
U.S. CDC IBV	97.86%	12,956	11,649	982	48	277

Oligonucleotides from each assay were assessed against IAV or IBV sequences obtained from GenBank on 29–30 March 2021. ^a^ Recall = true-positive (sum of the perfect match, single mismatch, and double mismatch)/(true-positive + false negative). ^b^ Failure represents IAV or IBV sequences that were not detected (false-negative).

## Data Availability

All code written in support of this publication is publicly available at https://github.com/jt-lanl/fever-probes.

## Supplementary Materials

The following are available online at www.mdpi.com/article/10.3390/bios11100367/s1, Figure S1, Hybridization kinetics of probes under varying reagent conditions. Figure S2, Specificity of IAV and IBV probes with varying RNA concentrations. Figure S3, Flow cytometry bead-based assay histogram results with the IAV FEVER probe and different synthetic targets. Figure S4, Flow cytometry data processing and gating strategies. Figure S5, Flow cytometry data processing for RNA match standard curve. Figure S6, Detectable probe after exonuclease digestion.
