# Supplementary material for: Optical Biosensor Platforms Display Varying Sensitivity for the Direct Detection of Influenza RNA
Source: Biosensors (Basel). 2021 Sep 30;11(10):367. doi: 10.3390/bios11100367 (PMC8534094; doi:10.3390/bios11100367)
Supplement: Supplementary file 1 [file biosensors-11-00367-s001.zip › biosensors-1385351-supplementary.pdf]

Supplementary Materials

Optical Biosensor Platforms Display Varying Sensitivity for the Direct Detection of Influenza RNA

Samantha J. Courtney [1], Zachary R. Stromberg [1], Adán Myers y Gutiérrez [2], Daniel Jacobsen [1], Loreen R. Stromberg [1], Kiersten D. Lenz [1], James Theiler [3], Brian T. Foley [4], Jason Gans [2], Karina Yusim [4] and Jessica Z. Kubicek-Sutherland [1,*]

[1] Physical Chemistry and Applied Spectroscopy, Los Alamos National Laboratory, Los Alamos, NM 87545, USA; sjc@lanl.gov (S.J.C.); zrs@lanl.gov (Z.R.S.); djacobsen@lanl.gov (D.J.); loreen@lanl.gov (L.R.S); kiersten@lanl.gov (K.D.L.)

[2] Biosecurity and Public Health, Los Alamos National Laboratory, Los Alamos, NM 87545, USA; adanm@lanl.gov (A.M.G.); jgans@lanl.gov (J.G.)

[3] Space Data Science and Systems, Los Alamos National Laboratory, Los Alamos, NM 87545, USA; jtheiler@lanl.gov

[4] Theoretical Biology and Biophysics, Los Alamos National Laboratory, Los Alamos, NM 87545, USA; btf@lanl.gov

* Correspondence: jzk@lanl.gov; Tel.: +1-505-665-6267

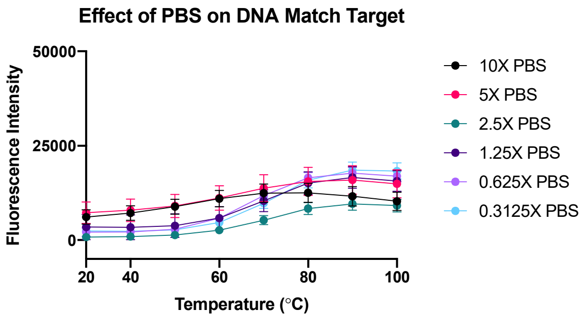

(**a**)

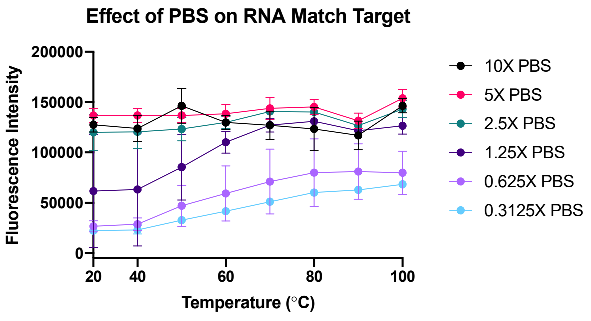

(**b**)

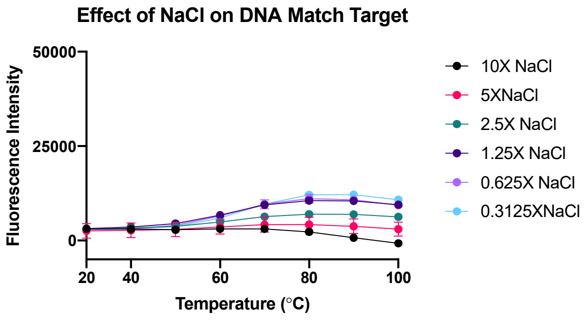

(**c**)

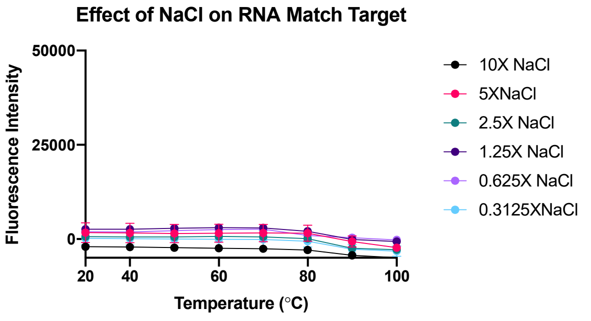

(**d**)

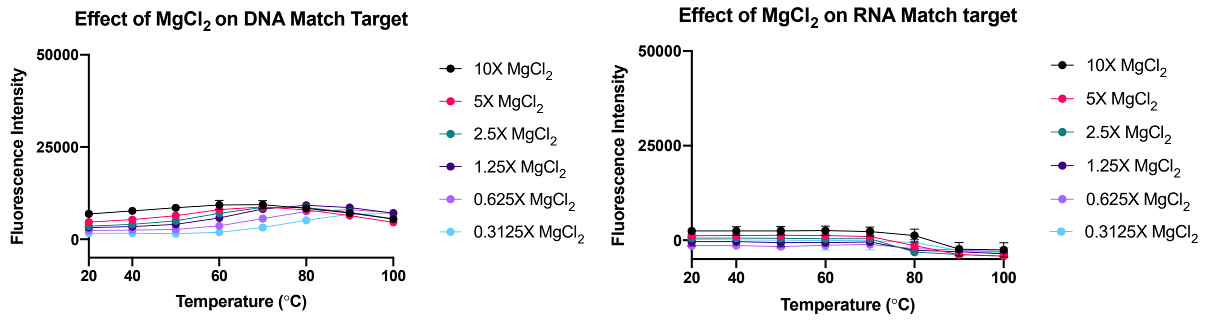

(e) (f)

Figure S1. Hybridization kinetics of probes under varying reagent conditions. Influenza A FEVER probe (200 nM) and its synthetic DNA match target and RNA match target (50 nM each) were tested under varying PBS, NaCl, and $MgCl_2$ conditions and were observed on a thermocycler via melt curves in separate 50 µL reactions: **(a)** Influenza A FEVER probe and synthetic DNA match target under varying PBS conditions; **(b)** Influenza A FEVER probe and synthetic RNA match target under varying PBS conditions; **(c)** Influenza A FEVER probe and synthetic DNA match target under varying NaCl conditions; **(d)** Influenza A FEVER probe and synthetic RNA match target under varying NaCl conditions; **e)** Influenza A FEVER probe and synthetic DNA match target under varying $MgCl_2$ conditions; **f)** Influenza A FEVER probe and synthetic RNA match target under varying $MgCl_2$ conditions.

Optimization of probe-target hybridization conditions

The FEVER probes did not detect their DNA match and mismatch targets as well as their RNA match and mismatch targets, so several nucleic acid stabilizing reagents were introduced into the reaction to address whether the signal to noise ratio would be improved in the presence of a buffer. Several reagents (PBS, NaCl, $MgCl_2$) were analyzed in serial dilutions in the presence of IAV FEVER probe to target hybridization. The effect of PBS at all concentrations on the probe to DNA match target hybridization increased the background noise and decreased the signal, which rendered the background signal (70 °C) higher than the expected target hybridization signal (25 °C) (Figure S1a). PBS increased the signal detection for probe to RNA match target hybridization, but the signal to background noise was indistinct and unspecified (Figure S1b). Therefore, PBS did improve the signal from the background noise. For all concentrations of NaCl, the signal for probe to DNA match target hybridization was lower than the background (Figure S1c). The signal for probe to RNA match target hybridization in the presence of NaCl was higher than the background, but the fluorescence intensity was dampened altogether (Figure S1d). Thus, NaCl did not improve the signal to background noise ratio. In a similar way, $MgCl_2$ did not optimize either target hybridization and dampened the overall fluorescence intensity (Figure S1e,f). Overall, the addition of certain reagents did not necessarily improve the probe to target hybridization signal to noise ratio. Thus, regardless of buffer conditions, the FEVER probes hybridized better to RNA match and mismatch targets than the DNA match and mismatch targets.

Table S1. Sequences of the synthetic DNA match and single base pair mismatch target sequences.

FEVER Probe	DNA Target Sequences
IAV	Match: TAAGCAAAACCCAGGGATCATTAATCAGGCACTCCTCAATTGC<br>Mismatch: TAAGCAAAACCCAGGGATC**G**TTAATCAGGCACTCCTCAATTGC
IBV	Match: TTTGGACCATCCATGCTATGGTTCTTGGCATTCCCTCAATTAC

Mismatch: TTTGGACCATCCATGCTATG**<u>T</u>**TTCTTGGCATTCCCTCAATTAC

The single mismatch base of the mismach RNA target sequences is **<u>bold</u>**.

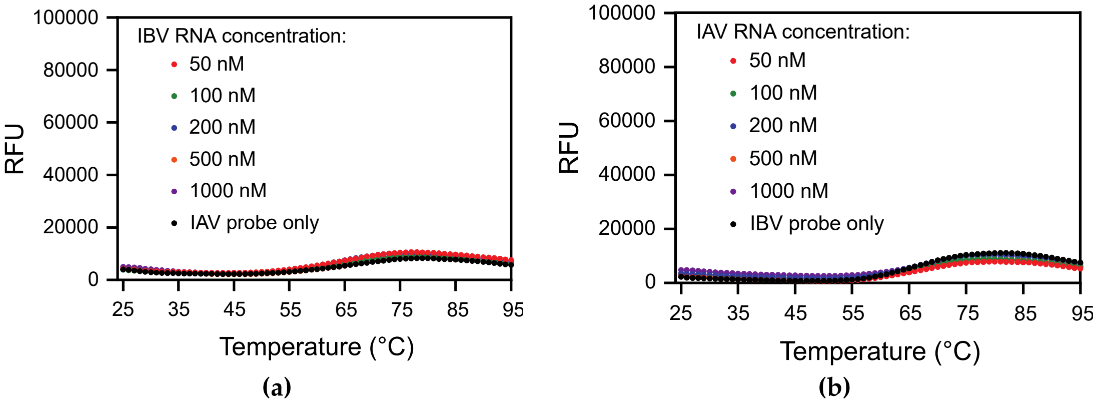

Figure S2. Specificity of IAV and IBV probes with varying RNA concentrations. (a) IBV RNA (0–1000 nM) was not detected using the 200 nM IAV probe. **(b)** IAV RNA (0–1000 nM) was not detected using the 200 nM IBV probe. Measurements were taken using a thermal cycler without amplification. RFU, relative fluorescence units.

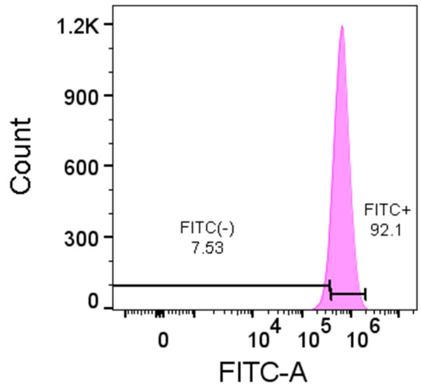

(**a**)

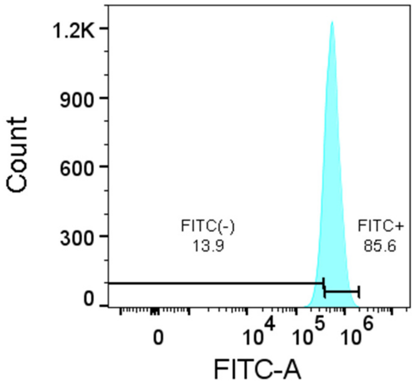

(b)

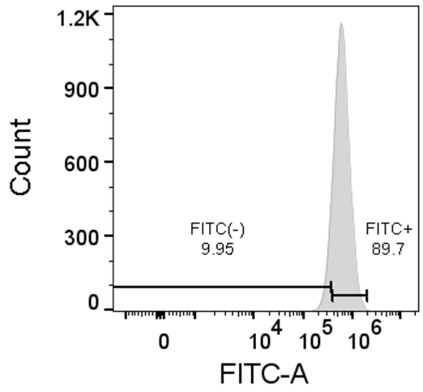

(c)

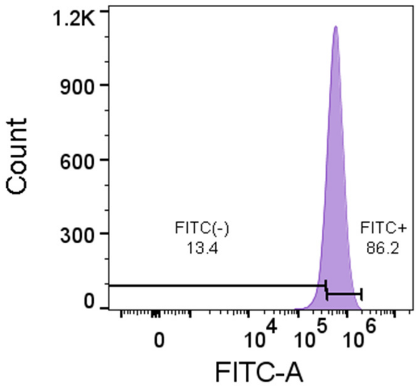

(d)

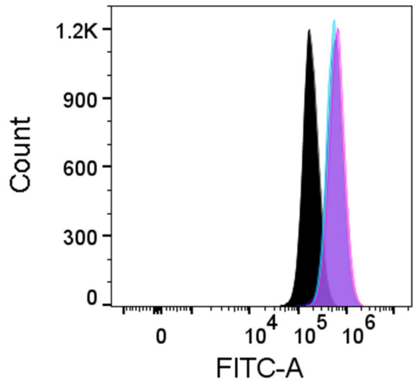

(e)

Sample Name	Count
S4 DNA Match	19705
S6 RNA Match1	19688
S5 DNA Mismatch	19575
S7 RNA Mismatch	19659
S3 Beads and Beacon	19736

(f)

Figure S3. Flow cytometry bead-based assay histogram results with the IAV FEVER probe and different synthetic targets. (a) DNA match target hybridization detection; **(b)** RNA match target hybridization detection; **(c)** DNA mismatch target hybridization detection; **(d)** RNA mismatch target hybridization detection; **(e)** All target detection histograms overlayed with the negative control "Beads and Beacon;" **(f)** Sample legend.

Flow cytometry assessment of MB probe hybridization to RNA, DNA, match and mismatch targets

The influenza A FEVER probe was analyzed in a streptavidin bead-based flow cytometry assay in the presence of its DNA match, DNA mismatch, RNA match, and RNA mismatch synthetic targets. In the presence of the DNA match target, the probe signal was 92.1 % positive in the FITC (+) gate (Figure S2A). In the presence of the RNA match target, the probe signal was 85.6 % positive in the FITC (+) gate (Figure S2B). In the presence of the DNA mismatch target, the probe signal was 89.7 % positive in the FITC (+) gate (Figure S2C). In the presence of the RNA mismatch target, the probe signal was 86.2 % in the FITC (+) gate (Figure S2D). When these histograms were overlayed with the negative control, the target hybridization was distinguishable (Figure S2E). Overall, probe to target hybridization detection was distinct from the negative control for both DNA and RNA target and was therefore a successful detection platform using the influenza A FEVER probe.

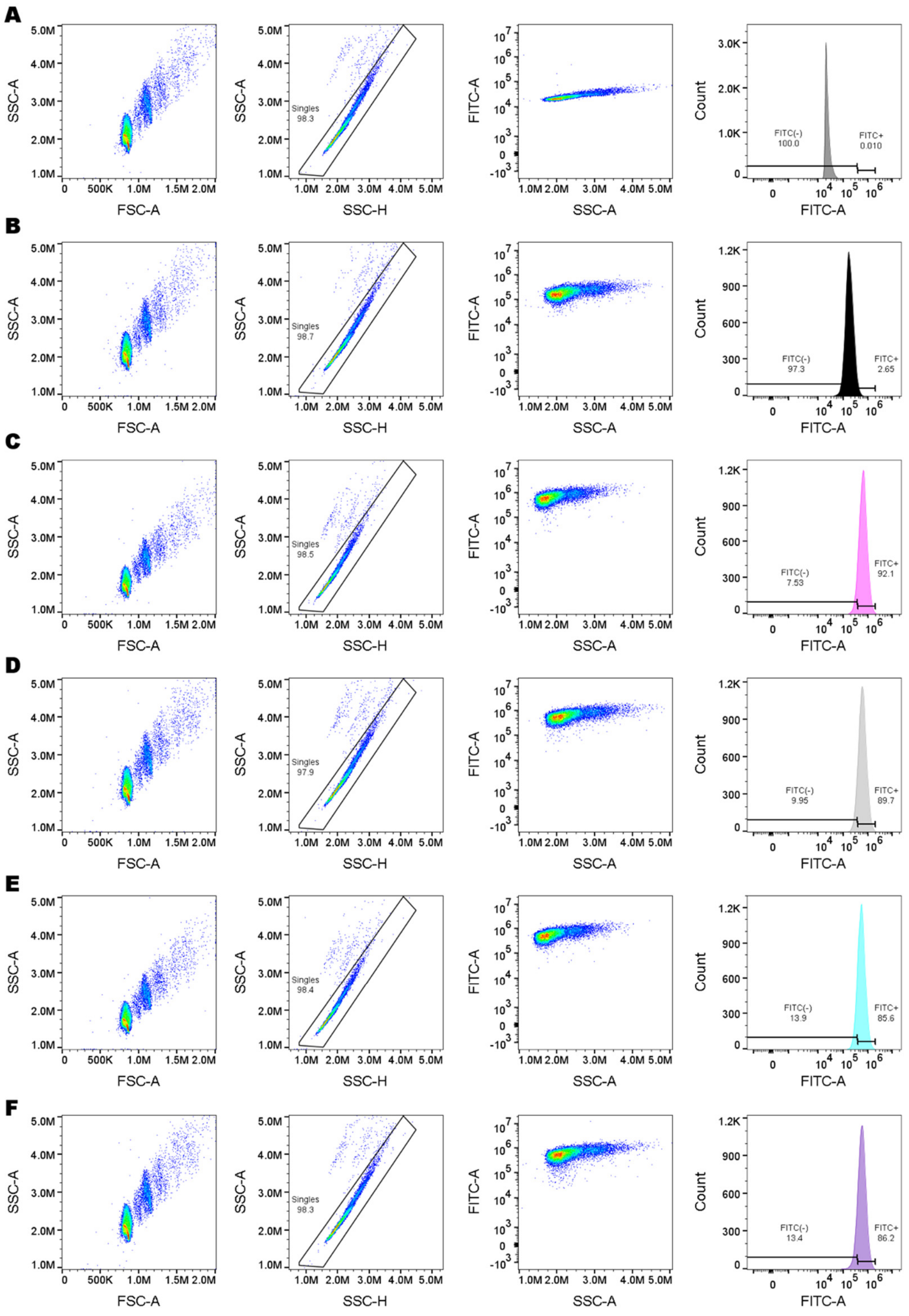

Figure S4. Flow cytometry data processing and gating strategies. (a) Left to right: SSC-A vs FSC-A plot of streptavidin control bead sample. Ungated sample view in SSC-A vs. SSC-H to identify and draw singles gate applied to all subsequent samples. Gated singles FITC-A vs. SSC-A pseudocolor plot used to identify the FITC (-) gate. Histogram plot of counts in the FITC (-) gate. FITC (+) gate also is displayed to demonstrate no counts are present. **(b)** Left to right: SSC-A vs. FSC-A plot of negative control beacon sample on streptavidin-coated particles. Singles gate established on streptavidin-coated particles is displayed in the SSC-A vs. FSC-A plot. Gated singles FITC-A vs. SSC-A pseudocolor

plot used to identify the FITC (-) gate. Histogram plot of counts in the FITC (-) gate. Some of the control beacon sample (2.65%) also presents in the FITC (+) gate indicating some low level fluorescence is associated with the beacon sample. **(c)** Left to right: SSC-A vs. FSC-A plot of DNA match sample. 98.5% of collected events fall within the established singles gate displayed in the SSC-A vs. FSC-A plot. Gated singles FITC-A vs. SSC-A pseudocolor plot used to identify the FITC (+) gate. Histogram plot of counts (92.1%) in the FITC (+) gate. FITC (+) gate demonstrates slightly less than an order of magnitude shift above the FITC (-) gate which causes some overlap of the two fluorescence peaks. **(d)** Left to right: SSC-A vs. FSC-A plot of DNA mismatch sample. 97.9% of collected events fall within the established singles gate displayed in the SSC-A vs. FSC-A plot. Gated singles FITC-A vs. SSC-A pseudocolor plot. Histogram plot of counts (89.7%) in the FITC (+) gate. **(e)** Left to right: SSC-A vs. FSC-A plot of RNA match sample. 98.4 % of collected events fall within the established singles gate displayed in the SSC-A vs. FSC-A plot. Gated singles FITC-A vs. SSC-A pseudocolor plot. Histogram plot of counts (85.6%) in the FITC (+) gate. **(f)** Left to right: SSC-A vs. FSC-A plot of RNA mismatch sample. 98.3 % of collected events fall within the established singles gate displayed in the SSC-A vs. FSC-A plot. Gated singles FITC-A vs. SSC-A pseudocolor plot. Histogram plot of counts (86.2%) in the FITC (+) gate. FITC (+) peaks all appear as consistent intensity counts between the positive samples, which is demonstrated by the histogram overlay plot seen in Figure S4H.

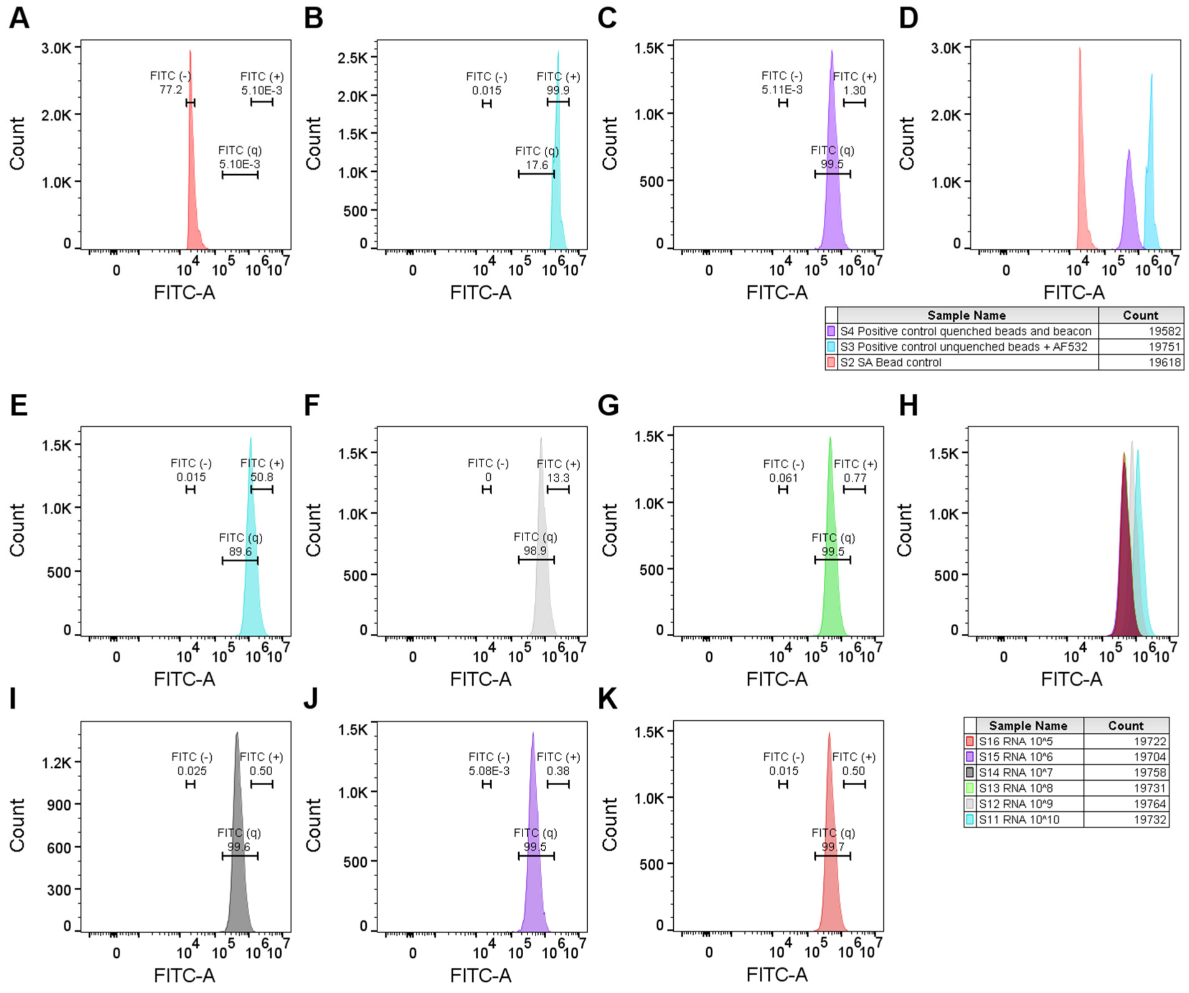

Figure S5. Flow cytometry data processing for RNA match standard curve. (a) FITC (-) gate established using negative control streptavidin-coated particles. **(b)** Positive control biotinylated particles coated with streptavidin AlexaFluor 532. **(c)** Positive control particles with beacon quenched with BHQ-1 quencher to establish a region of decreased

fluorescence intensity for the probe only "FITC (q)" gate. **(d)** Overlaid histogram of three control peaks used for gating the experiment. **(e)** Sample with 10 nM RNA falls both in the probe + RNA "FITC (+)" and FITC (q) gates indicating a strong positive signal. **(f)** Sample with 1 nM RNA is present both in the FITC (+) and FITC (q) gates, but with reduced intensity compared to the 10 nM RNA sample. **(g)** The 100 pM RNA sample falls almost entirely in the FITC (q) gates with decreased signal intensity, but still positive compared to the negative controls. **(h)** Overlay histogram of all RNA samples between 0.1 pM to 10 nM. **(i)** Sample with 10 pM RNA. **(j)** Sample with 1 pM. **(k)** Sample with 0.1 pM.

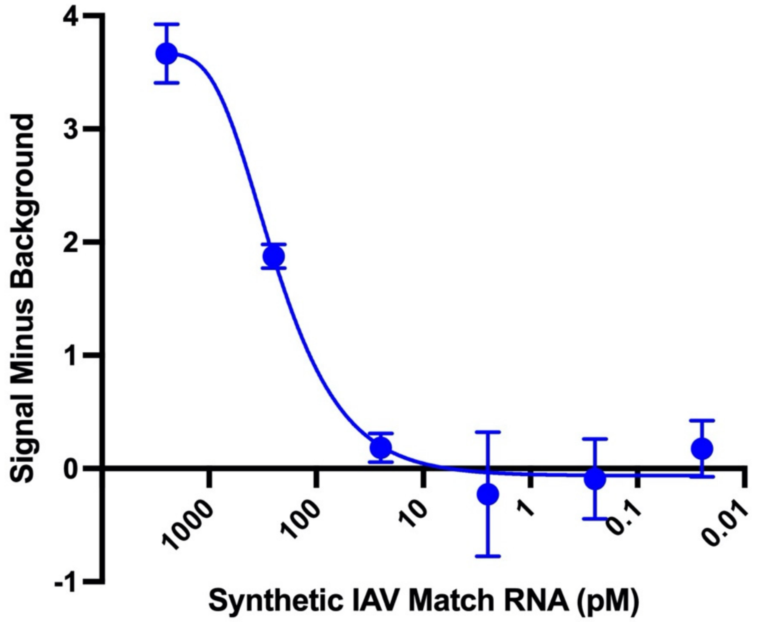

Figure S6. Detectable probe after exonuclease digestion. After exonculease digestion, remaining probe was measured via PCR. Background was the amount of probe measured by PCR for samples with no synthetic IAV match RNA added. Bars represent a 95% confidence interval.
